# Psoriasis Palmaris Cured by Iodide of Arsenic

**Published:** 1853-01

**Authors:** J. M. Sanford


From the Western Medico-Chirurgical Journal.

*Psoriasis Palmaris Cured by Iodide of Arsenic, by* J. M. Sanford, M. D.

Mr. J. Reed, of this city, merchant, aged 38 years, of sanguine temperament and vigorous constitution, applied to me, some time during the last July, on account of a disagreeable and troublesome affection of the hands and feet, with which he had suffered for the previous five years. Upon examination we found the palmar surface of both hands, wrists, and the heels of both feet covered with thick dense epidermal scales, very dry and hard, with deep fissures or cracks extending in the direction of the natural furrows of the palm and fingers. The hands and feet were extremely stiff and painful, and any attempt to use them suddenly, was attended with an increase of pain, and bleeding from the cracked portion of the diseased surfaces. There was also a very troublesome pricking or tingling sensation in the parts, and a burning or itching that much annoyed the patient. The eruption in question made its appearance by one or more inflamed and painful spots, of a dull red color, which extended in various directions, frequently coalescing. After a short time these patches became covered with scales, which increased in number and thickness until the epidermis seemed thick and hard as leather, and cracked as above-mentioned. Occasionally when these inflamed spots occurred in exposed situations they would suppurate and thus augment the suffering and discomfort of the patient. The discharge from these little abscesses was thin and sanious, and after its escape the spots seemed disposed to heal without those further changes, described as occurring when suppuration did not take place.

Inquiries were made relative to the influence of season upon the development of the disease, but it did not appear that it underwent any great change at any particular time. The eruption had been constantly present, to some extent, for five years, and although partially disappearing occasionally, these periods of partial exemption had no connection, as far as the patient was aware, with the season or with his habits of life.

He had applied to numerous physicians, from whom he had doubtless taken the usual remedies, and had also used various popular medicines, but without relief.

The *cause* of the disease, in this case, was obscure. There was no constitutional indisposition, nor anything in the patient's occupation or habits, that could act as a local irritant in its production. His general health was remarkably good; he had not suffered a functional disturbance for months, and presented, when I saw him, otherwise than in the respect mentioned, a perfect physiological condition.

I commenced the treatment by ordering him to drink freely of cream tartar, until the bowels were moved, and to apply at night a bread and milk poultice to the diseased parts. The poultices—which he had frequently applied, always relieved the heat and pricking of the parts, and softened the thickened epidermis, so that he could scrape away considerable portions after its removal. During the day he kept a cloth to the feet, wet in a strong solution of the Bicarb. of Soda, and frequently washed the hands in the same.

I was led to the employment of Iodide of Arsenic, by accident. I had intended, after a brief preparatory treatment, to place the patient on a more energetic course, and had thought of the Liquor Hydriodatis Arsenici et Hydrargyri, as the medicine to be used, as recommended by Dr. Graves, of Dublin. Finding myself unable to procure this at our drug stores, and having much confidence in the combination of Iodine and Arsenic in the treatment of various cutaneous diseases, I resorted to the Iodide of Arsenic.

Eight grains of this medicine were dissolved in four ounces of distilled water. Of this solution, twenty drops were given three times a day, in a few drachms of sweetened water. At the same time, fifteen grains of the same medicine was thoroughly incorporated with one ounce of simple cerate, a small portion of which was well applied to the diseased surfaces every morning after the removal of the poultice. The parts were thus in a soft state, admitting, without pain, the removal of the thickened cuticle, after which they were in good condition to receive the ointment.

This treatment was followed by the most decided and happy results. In two weeks the patient was very much improved, and in six weeks from the time it was commenced, no vestige of the disease remained. Two months have elapsed since the cure appeared complete and there is no return of the disease. This being the first thorough disappearance of the eruption in five years, together with its exemption from the modifying influences of season, encourage me to believe that the cure is permanent.

The prompt and decided effects of the Iodide of Arsenic in a case calculated to test its curative powers, justifies the inference that it is a remedy of peculiar efficacy in the treatment of the various forms of Psoriasis. No disagreeable local or constitutional effects followed its use.

I am at this time employing the same remedy in a protracted case of Ptyriasis, with the prospect of a similar happy result.

